# Burnout, Perceived Efficacy, and Job Satisfaction: Perception of the Educational Context in High School Teachers

**DOI:** 10.1155/2019/1021408

**Published:** 2019-04-09

**Authors:** María del Mar Molero Jurado, María del Carmen Pérez-Fuentes, Leonarda Atria, Nieves Fátima Oropesa Ruiz, José Jesús Gázquez Linares

**Affiliations:** ^1^Department of Psychology, Faculty of Psychology, University of Almería, 04120 Almería, Spain; ^2^Department of Psychology, Faculty of Psychology, Universidad Autónoma de Chile, 4780000 Santiago, Chile

## Abstract

Burnout is closely related to personal and contextual variables, especially job satisfaction and commitment, and other less studied psychological variables, such as perception of teaching efficacy or educational context.* Objective*. The general objective of this study was to examine the relationships of burnout with perceived educational context, perceived teaching efficacy (personal and collective), and job satisfaction and commitment.* Materials and Methods*. A battery of instruments was administered to 500 high school teachers at different schools in several Italian provinces.* Results*. The cluster analysis found that one-third of high school teachers had high burnout. Evidence was also found associating elevated burnout with low scores in perceived efficacy (personal and collective), low job satisfaction, and low professional commitment. Furthermore, perception of the educational context is less positive when the teachers experience high levels of burnout. Finally, the results showed the mediating effect of perceived personal efficacy on the relationship between burnout and job satisfaction.* Conclusions*. The results are discussed from the perspective of developing teaching autonomy on improving personal efficacy, decreasing burnout, and increasing job satisfaction in an educational system which reinforces individual and collective competence.

## 1. Introduction

The burnout syndrome is characterized by dealing with a range of symptoms related to psychophysical exhaustion, impaired relations, and professional inefficacy and with disillusion [1–3]. Some authors have shown that problematic behavior of students and dealing with their families are two main factors affecting job stress in teachers [4, 5]. At least 30% of teachers have experienced burnout in the last two decades with similar incidence in different countries, which has been accompanied by negative consequences to health, economic level, and degree of commitment and satisfaction in the teaching profession [6]. In this study, one of our objectives was to find homogeneous groups of teachers by burnout level. Then we analyzed the state of burnout in the high school context and its relationship with perceived educational context, teaching efficacy, job satisfaction, and commitment.

In the literature on the burnout syndrome, there is evidence of a significant relationship between burnout and perceived educational context [7–9]. The conclusions of the study by Khani and Mirzaee [7], for example, showed that contextual variables could not only cause burnout in teachers directly but also influence it indirectly by increasing the effect of different stressors. Similarly, research by Klusmann et al. [8], with a large sample of high school teachers from over one hundred German schools, revealed that when the analysis controlled for individual teacher characteristics, disciplinary problems in the classroom predicted a higher level of emotional exhaustion. In this vein, problems of coexistence such as fighting and problems with the teaching staff were also the aspects of most concern to the families in different European countries [10] Emotional exhaustion is associated with higher levels of nonphysical violence [11]. In another line, research by Skaalvik and Skaalvik [12] with Norwegian teachers showed that emotional exhaustion was related more strongly to pressures of time, while depersonalization and the reduced personal accomplishment were associated more intensely with parent-teacher relationships. Meanwhile, in a study by Hultell and Gustavsson [9], work demands (unsatisfied expectations, work load, the role of stress, routinization, social isolation, and passive coping strategy) were more closely related to burnout than work resources (autonomy, social support from colleagues, social support from supervisor or principal, satisfaction with salaries, mastery of skills, and active coping strategies) which were more connected to job commitment. García-Arroyo and Osca [13] suggested that burnout coping strategies should be based on nonlinear interactive models, since coping operates in a combined process in which some strategies affect others.

Interest in studying the relationship of burnout and teaching efficacy has been growing with time [14]. However, because teaching efficacy has been conceptualized and measured in different ways in different studies, some authors have preferred to differentiate between personal and collective efficacy [15–19]. Personal efficacy refers to confidence in one's own actions to reach expected results [15] and collective efficacy is defined rather as the belief in the ability of the school or team of teachers to perform actions leading to achievement of goals [17–19].

With regard to self-efficacy or personal efficacy and burnout, the empirical literature reflects a significant negative relationship between the two variables [20–24]. Thus Briones et al. [20] found that self-efficacy of teachers was a direct predictor of personal accomplishment, as well as perception of support received from colleagues. In this same direction, Evers et al. [21] found that beliefs of self-efficacy were related significantly and positively to personal accomplishment and, furthermore, had a significant negative relationship with the emotional exhaustion and depersonalization dimensions. Ventura et al. [24] found that employees with more self-efficacy at work perceived more challenging demands and fewer impediments, and this in turn was related to stronger commitment and less burnout. Similarly, in healthcare, Molero et al. [23] found that self-efficacy and stress management protected from burnout. In this area, it was the emotional intelligence dimension of adaptability which was the strongest predictor of self-efficacy [25]. Higher levels of self-esteem have also been linked to lower levels of burnout [26]. However, less research has been done on the relationship between collective self-efficacy and burnout and there are no significant results. For example, in the study by Malinen and Savolainen [22] with a sample of Finnish high school teachers, collective efficacy concerning how discipline was maintained among students did not explain burnout.

There is plentiful precedent literature supporting the assumption that job dissatisfaction and burnout maintain a close relationship with each other [27–29]. Thus in the study by Skaalvik and Skaalvik [12], teachers' job dissatisfaction was directly related to emotional exhaustion and diminished personal accomplishment. On the contrary, greater teacher job satisfaction at different grade levels was related to satisfaction of the psychological needs for autonomy, teaching staff competence, and relations [24], with self-determined motivation [24, 30], with cognitive self-regulation [31], ans with stronger social support [32]. Furthermore, job satisfaction has been found empirically to have a positive role in subjective wellbeing [33, 34] and teacher self-concept [35].

The perception of the educational context also has an important role in job satisfaction [7, 12, 35]. Job satisfaction has been found to be indirectly related to all the aspects of the school context (support from supervision, pressure of time, relationships with parents, and autonomy), through emotional exhaustion and reduction in personal accomplishment [12]. Other studies have related job satisfaction, burnout, and teaching efficacy. For example, Skaalvik and Skaalvik [36] found that teacher self-efficacy and the two dimensions of teacher burnout (emotional exhaustion and depersonalization) were significantly associated with teacher job satisfaction. Briones et al. [20] demonstrated that teacher self-efficacy was an indirect predictor of job satisfaction.

With respect to the relationship between job satisfaction and collective efficacy, several different studies have found that collective efficacy is not correlated with teacher job satisfaction [22, 36]. Since the role of efficacy in job satisfaction and burnout is a line of research where the most studies, at least in education, have been suggested, another of our major objectives was to analyze the mediating role of teaching efficacy in the relationship between burnout and job satisfaction.

In addition, with respect to the relationship between burnout, commitment, and educational context, job commitment seems to modulate the relationship between demands and burnout and between resources (personal and related to the job) and burnout [37]. Meanwhile, Pérez-Fuentes, Molero, Gázquez, and Oropesa [38], with a large sample of healthcare professionals, found that the interpersonal dimension of emotional intelligence was the strongest predictor of job commitment. Likewise, commitment was found to be positively associated with self-efficacy and negatively with burnout [39].

After analyzing the most relevant findings of previous studies on burnout in the high school context and its relationship with different contextual and personal variables, the main objectives and hypotheses of this study are discussed below. As mentioned a few paragraphs above, one of the purposes of this study was to find homogeneous groups of teachers by their level of burnout. It was also intended to examine the relationships of burnout with both perception of the educational context, perceived teaching efficacy (personal and collective), and job satisfaction and commitment. In this sense, we also wanted to find any significant differences between the high and low burnout groups based on the variables above. Finally, we attempted to take a further step forward by exploring the mediating role of perceived teaching efficacy on the relationship between burnout and job satisfaction.

## 2. Materials and Methods

### 2.1. Participants

The study sample consisted of 500 7th and 8th grade high school teachers, selected at random from different schools in the Sicilian provinces of Trapani, Agrigento, and Palermo (Italy). Of this sample, 67.2% (*n*=336) were women and 32.8% (*n*=164) were men. The most representative group of 40.4% were teachers aged 46 to 55 (*n*=202), followed by the group from 36 to 45 years who made up 27.2% (*n*=136) of the sample, those who were over 55 comprised 22.6% (*n*=113), and finally, the least representative group of 9.8% of the sample were the youngest, from 25 to 35 years. By level of education, 84.2% (*n*=421) had undergraduate degrees, 13% (*n*=65) had Master's degrees, 2.4% (*n*=12) had diplomas, and 0.4% (*n*=2) had Ph.D. degrees (Table 1). 

Concerning their professional characteristics, the groups of teachers with seniority (years of experience) of 11 to 20 years and from 21 to 30 years made up 33.8% (*n*=169) and 25% (*n*=125) of the sample, respectively. By type of contract, 73.4% (*n*=362) had a permanent contract and 27.6% (*n*=138) temporary. Finally, although all the teachers in the sample taught high school, 48.8% (*n*=244) taught seventh grade and 51.2% (*n*=256) eighth grade.

### 2.2. Instruments

#### 2.2.1. LBQ: Link Burnout Questionnaire [3]

This is a self-report questionnaire which provides new burnout indicators for those who work in service professions. Santinello [3] reviewed the three dimensions studied by the MBI and added the new disillusion scale to enlarge the theoretical tradition of burnout.

The four dimensions examined by the LBQ are (1) psychophysical exhaustion, (2) impaired relations, (3) professional inefficacy, and (4) disillusion. Disillusion is manifested by loss of passion and enthusiasm for daily activities. Burnout may therefore be characterized as the final state in a long process of disillusion.

The LBQ consists of 24 items with a six-point Likert-type response scale for study of four dimensions, each with three positive and three negative elements: the psychophysical dimension (energy-exhaustion), relationships (involvement-deterioration), professional competence (efficacy-inefficacy), and existential expectations (satisfaction-disillusion). The internal consistency of the scales, according to data found by the author, varies from .68 (professional inefficacy) to .85 (disillusion). In our case, the Cronbach's alpha for the complete questionnaire was .89 and for each one of the scales it was psychophysical exhaustion (*α*=.70), impaired relations (*α*=.66), professional inefficacy (*α*=.65), and disillusion (*α*=.82).

#### 2.2.2. Assessment Questionnaire for Convictions about Efficacy, Perceived Context, Job Attitudes, and Satisfaction in School Contexts [19]

This questionnaire is comprised of several scales with a seven-point Likert response scale: perceived personal efficacy scale (the teacher's conviction of being up to the demands of their role and coping with any emergency or eventuality, for example, with regard to families or their colleagues, in managing the class or problem students) and perceived collective efficacy scale (the teacher's beliefs with regard to the ability of the school to dominate complicated tasks and cope with innumerable critical situations, approach problems related to school quitting, manage relations with local authorities, and face the demands of school autonomy).

Job satisfaction is the degree of satisfaction with the role, possibilities for personal growth, and work environment and the degree to which personal needs are satisfied by the job.

Job commitment is bond which the person establishes with the organization and their commitment to achieve objectives.

Scales concerning the perceived educational context are the principal perception scale (degree to which the teachers evaluate the principal's ability to identify resources within the school, promote cooperation, and set clear objectives), colleague perception scale (perception of job relations, work of fellow teachers, and efficacy of communication among colleagues), student perception scale (perception of student-teacher relations, students' interest in the subjects taught, and respect for the setting and persons), family perception scale (perception of parent-teacher relations, degree of parent participation, and interest in their children's school life), technical-auxiliary staff scale (perception of how technical-auxiliary staff works in terms of competence and flexibility), physical environment perception scale (evaluation of the school's facilities, adequacy for the educational demands, and general safety). Its reliability was calculated using the Cronbach's alpha coefficient, which varied from .90 (job satisfaction) to .95 (perception of the school principle), and a value of .98 for the complete questionnaire.

### 2.3. Procedure

After consent was received from the direction of the participating schools, a meeting was held with the teachers where the study was presented, explaining its importance and clarifying its objectives, to acquire the approval and participation of all the teachers. The questionnaire was given directly to each teacher for completion at will. Participation in the study was voluntary and anonymity of participants was guaranteed. A period of one to two weeks was set for compilation of the data. It was administered at the schools in the middle of the school year. The SPSS version .23 for Windows was used for data processing and analysis.

#### 2.3.1. Data Analysis

First, bivariate correlations were done to explore the relationships between variables. Then two-stage cluster analysis was done to find the groups of professionals based on their scores in the burnout dimensions. When the groups or clusters had been identified, a comparison of means was done using the Student's* t* test for independent samples to determine the existence of any significant differences between burnout groups with respect to their perception of the educational context, perceived efficacy (personal and collective), commitment, and job satisfaction.

Finally, to compare the mediating effect of the perceived efficacy variables, a multiple mediation analysis was performed with two chained mediators. The macro for SPSS by Preacher & Hayes [40, 41] was used for computation of the mediation model. Bootstrapping was applied with coefficients estimated from 5000 bootstrap samples.

## 3. Results

### 3.1. Burnout in High School Teachers

**Figure 1:**
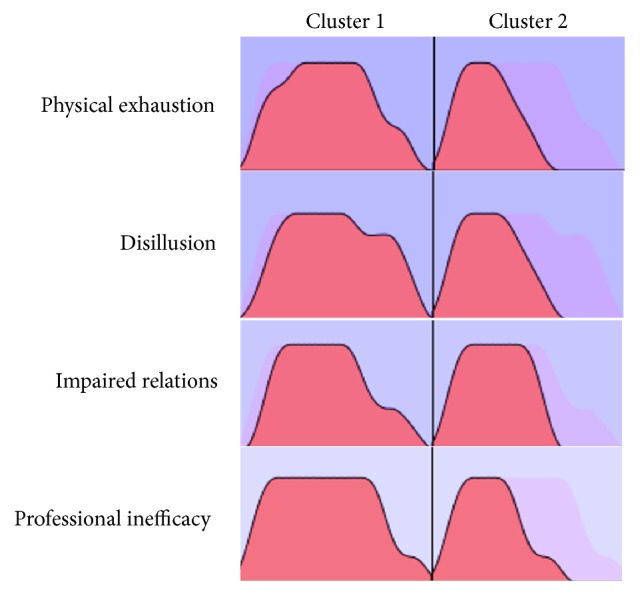
Cluster composition (*N*=500).* Note.* Factors are in the order of importance of input.

**Figure 2:**
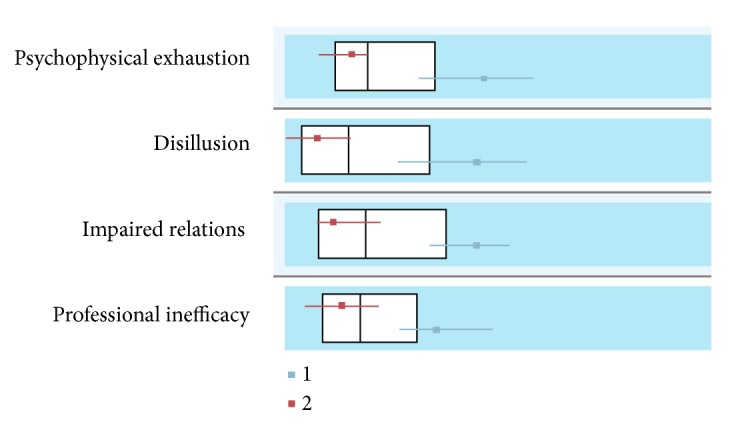
Comparison of clusters (*N*=500).

A two-stage cluster analysis was done with the burnout factors to form the groups (Figures 1 and 2). Two groups resulted from inclusion of these variables with the following distribution: 32.6% (*n*=163) of subjects in Cluster 1 and 67.4% (*n*=337) in Cluster 2.  Table 2 summarizes the means of evaluations of different aspects of the educational context for the total sample of teachers and for each of the clusters.

The first group resulting from the cluster analysis (Cluster 1) was characterized by scores above the mean for the total sample in psychophysical exhaustion (*M*=17.83), impaired relations (*M*=17.48), professional inefficacy (*M*=14.78), and disillusion (*M*=17.67). Therefore, the subjects in this cluster were grouped together because of their high levels in all the burnout dimensions.

The second group (Cluster 2) grouped teachers with mean scores below those found for the total sample in all the burnout dimensions: psychophysical exhaustion (*M*=9.98), impaired relations (*M*=10.14), professional inefficacy (*M*=9.15), and disillusion (*M*=8.76). That is, those in Cluster 2 coincided in having scores below the mean on the burnout dimensions.

### 3.2. Burnout in High School Teachers and Its Relationship with Perception of the Educational Context, Efficacy, Commitment, and Job Satisfaction

As shown in Table 3, the four dimensions of burnout correlate negatively with the perception of the teachers of the educational context (the management team, colleagues, technical-auxiliary staff, secretarial staff, families, students, and physical school environment). Burnout was also found to be negatively correlated with personal and collective efficacy, with organizational commitment and job satisfaction.

A Student's* t* test for independent samples was carried out on the groups classified based on the two-cluster solution to find any differences between the clusters with respect to the rest of the variables analyzed. As observed in Table 4, there were significant differences between the groups with high and low burnout levels for all aspects related to teacher perception of educational context, efficacy, commitment, and job satisfaction, where Cluster 1 (burnout dimension scores above the sample mean) showed lower scores in all the dimensions analyzed.

### 3.3. Mediation Model for Estimating Predictors and Paths of Mediation Effects of Perceived Efficacy on Job Satisfaction

**Figure 3:**
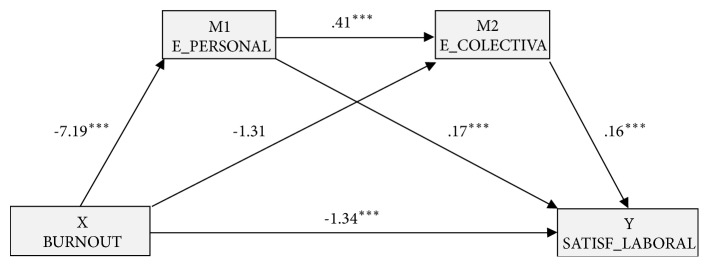
Multiple mediation model of perceived efficacy (personal and collective) on the relationship between* burnout* and job satisfaction.

Based on the results of the cluster analysis, the burnout groups (recoded: ▼Burnout=0; ▲Burnout=1) were taken as the independent or predictor variable and perceived efficacy (personal and collective) as the mediating variables. Thus the multiple mediation model was computed with two mediator variables (M_1_: E_pers_ and M_2_: E_colec_), with job satisfaction as the dependent variable (Figure 3).

In the first place, a statistically significant effect [B=-7.19,* p*<.001] of* burnout* (X) on perceived personal efficacy (M_1_) was observed. The second regression analysis took as the result variable Mediator 2 (perceived collective efficacy) and included burnout (X) and perceived personal efficacy (M_1_) in the equation. There was a significant effect of personal efficacy [B=.41,* p*<.001] on collective efficacy (M_2_), but not on burnout [B=-1.31,* p*=.137].

In the following regression analysis, the effect of the independent variable and of the two mediators was estimated taking job satisfaction as the result variable (Y). In all cases, significant effects were observed: personal efficacy [B=.17,* p*<.001], collective efficacy [B=.16,* p*<.001], and* burnout* [B=-1.34,* p*<.001]. The total effect of the model was also significant [B=-3.28,* p*<.001].

Finally, the analysis of indirect effects was carried out using bootstrapping, and data found supported a level of significance for Path 1 [ind_1_: X → M_1_ → Y; B=-1.22, SE=.27, 95% CI (-1.84, -.73)] and Path 2 [ind_2_: X → M_1_ → M_2_ → Y; B=-.49, SE=.13, 95% CI (-.78, -.28)]. In both cases perceived personal efficacy seemed to mediate the effect of burnout on job satisfaction. However, the indirect effect expressed in Path 3 [ind_3_: X → M_2_ → Y; B=-.21, SE=.16, 95% CI (-.55, .08)] was not significant.

## 4. Discussion

One of the first ideas inferred from the above analysis of burnout is that teachers with high and low burnout levels are clearly distinguished from each other. The percentages found in this study were distributed as follows: fewer teachers, around a third (32.6%), showed high burnout, while most of them, 67.4%, showed low levels. We can therefore respond to our first research hypothesis, by showing the significant prevalence of burnout among high school teachers, considering the severe risk to health this implies. The incidence is similar to what it was several decades ago, and in view of the characteristics of the postmodern society and the current education model, could increase in the coming years if opportune measures are not taken.

The main findings of our study regarding the correlation analyses of burnout and its relationship to the perceived educational context, teacher efficacy, job satisfaction, and commitment generally coincide with analyses in previous literature and show clear evidence that the dimensions of burnout examined (psychophysical exhaustion, impaired relationships, professional efficacy, and disillusion) strongly correlate with all the variables above.

Concerning the relationship of burnout to perceived educational context, in the first place, our data showed that there was a close association between the perception of students and psychophysical exhaustion, and vice versa. The results of Klusmann et al. [8], who found that disciplinary problems in the classroom predicted a higher level of emotional exhaustion, were along this same line. One possible explanation for these results is that teachers with high psychophysical exhaustion could be using more passive strategies to cope with job demands, while teachers with less psychophysical exhaustion were able to rely on personal resources which enabled them to cope actively with conflictive situations in the classroom and be more professionally committed [9]. In the second place, the results found showed a moderate relationship between perception of the director and disillusion. These results do not coincide with those of the study by Hultell and Gustavsson [9], in which the director's social support showed a stronger connection with job commitment than burnout. This result may suggest that job commitment plays an important role in the relationship between perceived educational context and burnout, since it seems to modulate the relationship between the demands and resources and burnout [37]. Other studies have found that high scores in job satisfaction were indirectly related to better perception of support with supervision by teaching staff through emotional exhaustion and lack of personal accomplishment [12, 35]. Therefore, job commitment and satisfaction could have a mediating role in the relationship between burnout and perceived educational context, specifically, in the dimension of perception of the director. In any case, the role of these variables in the perceived educational context and burnout should continue to be studied to progress in this line of research in the future.

Our data on the relationship between burnout and perceived personal efficacy reflected that there was a strong negative association between perceived personal efficacy and psychophysical exhaustion, coinciding with the results found in the study by Evers et al. [21]. In our study, perceived personal efficacy was significantly negatively associated with the professional burnout inefficacy and disillusion dimensions. Other studies point to the same direction [22, 24]. Furthermore, other researchers have found that lower perceived self-efficacy predicted higher burnout due to the lack of personal accomplishment [20, 21].

Results found in relation to the correlation between burnout and job satisfaction supported a strong relationship between the dimensions of burnout (psychophysical exhaustion, impaired relations, professional inefficacy, and disillusion) and job satisfaction. These data are coherent with those found by other authors [12, 27–29, 36].

Our study found that in the relationship between burnout and organizational commitment, job commitment was related moderately to psychophysical exhaustion, and more closely to disillusion. Other studies suggest, as mentioned above concerning the relationship between burnout and perception of the educational context, that job commitment must be considered a modulating variable in the relationship between perception of the educational context and burnout [37]. Ventura et al. [24] found that staff with the most self-efficacy at work perceived more challenging demands and fewer impediments, and this in turn was related to more commitment and less burnout. Studies in a healthcare context also support the existence of a significant negative correlation between burnout and job commitment [39], and others, where job commitment has been closely related to emotional intelligence [38], and high self-efficacy exerts a protector role against burnout [23].

Based on the findings mentioned, we can say that our second research hypothesis, in which we expected to find that teachers with high burnout would have higher scores in the variables analyzed (perception of the educational context, perceived efficacy, job commitment, and satisfaction), was fulfilled. Solid evidence was found demonstrating that the group of teachers with high burnout showed lower scores in perception of the educational context, professional efficacy, satisfaction, and job commitment. Most of these results coincide with those found in other studies on burnout and perception of educational content [7–9, 12], burnout and personal efficacy [20–24], burnout and job satisfaction [12, 27–29], and lastly, burnout and job commitment [37].

Finally, empirical data in this study back our third hypothesis, showing that perceived personal efficacy exerted a mediating effect in the relationship between burnout and job satisfaction; however, the same was not true of collective efficacy. These results are coherent with those found by other researchers. Skaalvik and Skaalvik [36] found that teacher self-efficacy was significantly and positively associated with job satisfaction. Briones et al. [20] discovered that teacher self-efficacy was an indirect predictor of job satisfaction. Furthermore, with regard to collective efficacy, some researchers have found that perceived collective efficacy did not explain job satisfaction [22, 36]. Therefore, personal factors, specifically perceived personal efficacy, that is, the confidence in one's own possibilities and abilities to resolve and perform teaching functions, has more weight in the relationship between burnout and job satisfaction than social and contextual factors, such as beliefs about school management.

Given the crucial role of the mediating effects of personal efficacy perceived in the relationship between burnout and job satisfaction, action should be taken to improve confidence in one's own possibilities, by setting realistic goals adjusted to personal skills and abilities, developing high expectations about one's own performance, cultivate emotional intelligence and positive emotions, increase mental flexibility and creativity, stimulating personal initiative, and finally, support reference models to orient professional performance, promoting learning from experience (learn by doing).

This study has some methodological limitations. It is a cross-sectional design with the limitations of such studies, so it would be advisable for this research to be accompanied by other longitudinal studies. The population subject of this study was made up of high school teachers, which must be taken into account when generalizing the results. With respect to the evaluation procedures, we should mention the limitations of exclusive use of self-reports for measuring burnout, which would have to be completed with the use of other measurement instruments (direct observation, interviews), and finally, the results should be replicated and tested in other countries and cultures to broaden their reliability and validity.

Based on this study new questions are posed, for example, the role of the motivational variables in personal efficacy and its relationship with job commitment and how perception of the educational context contributes to development of the subjective wellbeing (satisfaction with life and happiness) of teachers. By way of a synthesis, we offer some final reflections on possible improvement of future interventions for burnout and job satisfaction of high school teachers: (1) due to the importance of emotional and motivational factors in teaching-learning, a teacher awareness training program on adolescent development should be started up and active coping strategies for professional demands in education, increasing their resources for improving their teaching commitment and managing stress; (2) the teaching staff should acquire skills and abilities for classroom use of creative, participatory, and dialogical methodologies, through a close, continual, and extended consulting service; (3) contributing to teacher autonomy to improve the perception of personal efficacy through an education system which reinforces individual and collective competence.

## 5. Conclusions

The following conclusions may be arrived at the following. On one hand, a third of high school teachers have a high level of burnout. Our results show that when the teaching staff experiences high burnout levels, perception of the educational context is less positive. In this study, the high burnout level was associated with low scores in perceived efficacy (personal and collective), low job satisfaction, and low professional commitment.

In addition, this study demonstrates that perceived personal efficacy exerts a mediating effect on the relationship between burnout and job satisfaction. In view of these findings, we propose that burnout in high school teachers be prevented by reinforcing job satisfaction and increasing perceived teaching efficacy and that educational entities provide specific training adapted to the teaching staff and supervision, consulting, and extended support in student matters of their interest, to increase autonomy and perceived efficacy. The teaching staff should be given sufficient time and space to assimilate professional competences related to teaching. And finally, to increase job satisfaction, it is important for the teacher's work to be recognized by the educational community (family, school, students) and receive affective, social, and economic compensation in a balance between demands or requirements and results achieved.

## Figures and Tables

**Table 1:** Distribution of the sample by sociodemographic and professional variables.

	*n*	%
Sex		
Male	164	32.8
Female	336	67.2
Age		
From 25 to 35 years	49	9.8
From 36 to 45 years	136	27.2
From 46 to 55 years	202	40.4
Over 55 years	113	22.6
Education		
Diploma	12	2.4
Undergraduate degree	421	84.2
Master's degree	65	13.0
Ph.D.	2	0.4
Years of experience		
5 years or less	61	12.2
From 6 to 10 years	71	14.2
From 11 to 20 years	169	33.8
From 21 to 30 years	125	25.0
Over 30	74	14.8
Type of contract		
Permanent	362	72.4
Temporary	138	27.6
Teaching high school		
7th grade	244	48.8
8th grade	256	51.2

**Table 2:** Mean scores on burnout for the total sample (*N*=500) and clusters.

Burnout	Total sample (*N*=500)	Cluster	
		1 ▲ Burnout (*n*=163)	2 ▼ Burnout (*n*=337)
Physical exhaustion	*M* = 12.54 (*SD* = 4.99)	*M* = 17.83 (*SD* = 4.71)	*M* = 9.98 (*SD* = 2.47)
Impaired relations	*M* = 12.53 (*SD* = 4.93)	*M* = 17.47 (*SD* = 4.09)	*M* = 10.14 (*SD* = 3.24)
Professional Inefficacy	*M* = 10.98 (*SD* = 4.16)	*M* = 14.77 (*SD* = 4.28)	*M* = 9.14 (*SD* = 2.56)
Disillusion	*M* = 11.66 (*SD* = 5.77)	*M* = 17.67 (*SD* = 5.61)	*M* = 8.76 (*SD* = 2.88)

**Table 3:** Burnout, perception of the educational context, efficacy, commitment, and job satisfaction. Correlation matrix.

	1	2	3	4	5	6	7	8	9	10	11	12	13	14
1. Physical exhaustion	–													
2. Impaired relations	.61^*∗∗∗*^	–												
3. Professional inefficacy	.58^*∗∗∗*^	.55^*∗∗∗*^	–											
4. Disillusion	.66^*∗∗∗*^	.61^*∗∗∗*^	.64^*∗∗∗*^	–										
Perception of the educational context														
5. Management team	-.23^*∗∗∗*^	-.27^*∗∗∗*^	-.26^*∗∗∗*^	-.34^*∗∗∗*^	–									
6. Colleagues	-.21^*∗∗∗*^	-.20^*∗∗∗*^	-.29^*∗∗∗*^	-.28^*∗∗∗*^	.61^*∗∗∗*^	–								
7. Tech.-auxiliary staff	-.27^*∗∗∗*^	-.21^*∗∗∗*^	-.25^*∗∗∗*^	-.26^*∗∗∗*^	.53^*∗∗∗*^	.55^*∗∗∗*^	–							
8. Secretarial staff	-.24^*∗∗∗*^	-.12^*∗∗∗*^	-.25^*∗∗∗*^	-.26^*∗∗∗*^	.61^*∗∗∗*^	.49^*∗∗∗*^	.70^*∗∗∗*^	–						
9. Families	-.19^*∗∗∗*^	-.31^*∗∗∗*^	-.22^*∗∗∗*^	-.28^*∗∗∗*^	.52^*∗∗∗*^	.58^*∗∗∗*^	.48^*∗∗∗*^	.48^*∗∗∗*^	–					
10. Students	-.24^*∗∗∗*^	-.40^*∗∗∗*^	-.26^*∗∗∗*^	-.25^*∗∗∗*^	.45^*∗∗∗*^	.52^*∗∗∗*^	.39^*∗∗∗*^	.33^*∗∗∗*^	.71^*∗∗∗*^	–				
11. Physical environment	-.20^*∗∗∗*^	-.16^*∗∗∗*^	-.20^*∗∗∗*^	-.26^*∗∗∗*^	.50^*∗∗∗*^	.47^*∗∗∗*^	.47^*∗∗∗*^	.51^*∗∗∗*^	.47^*∗∗∗*^	.36^*∗∗∗*^	–			
12. Perceived personal efficacy	-.38^*∗∗∗*^	-.37^*∗∗∗*^	-.47^*∗∗∗*^	-.43^*∗∗∗*^	.47^*∗∗∗*^	.44^*∗∗∗*^	.40^*∗∗∗*^	.43^*∗∗∗*^	.37^*∗∗∗*^	.40^*∗∗∗*^	.33^*∗∗∗*^	–		
13. Perceived collective efficacy	-.18^*∗∗∗*^	-.21^*∗∗∗*^	-.23^*∗∗∗*^	-.26^*∗∗∗*^	.67^*∗∗∗*^	.63^*∗∗∗*^	.53^*∗∗∗*^	.55^*∗∗∗*^	.58^*∗∗∗*^	.53^*∗∗∗*^	.54^*∗∗∗*^	.43^*∗∗∗*^	–	
14. Organizational commitment	-.36^*∗∗∗*^	-.37^*∗∗∗*^	-.40^*∗∗∗*^	-.46^*∗∗∗*^	.56^*∗∗∗*^	.56^*∗∗∗*^	.43^*∗∗∗*^	.46^*∗∗∗*^	.49^*∗∗∗*^	.44^*∗∗∗*^	.47^*∗∗∗*^	.58^*∗∗∗*^	.54^*∗∗∗*^	–
15. Job satisfaction	-.42^*∗∗∗*^	-.36^*∗∗∗*^	-.41^*∗∗∗*^	-.46^*∗∗∗*^	.62^*∗∗∗*^	.64^*∗∗∗*^	.47^*∗∗∗*^	.49^*∗∗∗*^	.55^*∗∗∗*^	.49^*∗∗∗*^	.48^*∗∗∗*^	.59^*∗∗∗*^	.57^*∗∗∗*^	.75^*∗∗∗*^

Note. ^*∗∗∗*^*p*<.001

**Table 4:** Perception of the educational context, efficacy, commitment, and job satisfaction. Descriptive statistics and *t* test by *burnout* group.

	Cluster 1 ▲ Burnout			Cluster 2 ▼ Burnout			*t*	*p*
	*N*	*M*	*SD*	*N*	*M*	*SD*		
Perception of the educational context								
…management team	163	38.85	7.38	337	42.55	7.78	-5.06^*∗∗∗*^	.000
…colleagues	163	30.90	6.31	337	34.08	7.07	-4.86^*∗∗∗*^	.000
… technical-auxiliary staff	163	20.76	4.89	337	23.57	4.20	-6.30^*∗∗∗*^	.000
… secretarial staff	163	11.00	2.72	337	12.21	2.18	-4.94^*∗∗∗*^	.000
… families	163	18.69	4.92	337	21.07	4.87	-5.08^*∗∗∗*^	.000
…students	163	19.19	4.84	337	21.77	4.51	-5.85^*∗∗∗*^	.000
…physical environment	163	19.02	5.73	337	21.65	5.07	-4.98^*∗∗∗*^	.000
Perceived personal efficacy	163	65.73	9.80	337	72.92	8.29	-8.06^*∗∗∗*^	.000
Perceived collective efficacy	163	47.06	8.48	337	51.38	9.79	-4.82^*∗∗∗*^	.000
Organizational commitment	163	31.60	7.50	337	36.70	5.94	-7.60^*∗∗∗*^	.000
Job satisfaction	163	21.93	4.87	337	25.22	3.39	-7.74^*∗∗∗*^	.000

Nota. ^*∗∗∗*^*p*<.001

## Data Availability

The data used to support the findings of this study are available from the corresponding author upon request.

## References

[1] Maslach C., Jackson S. E. (1981). The measurement of experienced burnout. *Journal of Organizational Behavior*.

[2] Pérez-Fuentes M. D. C., Gázquez Linares J. J., Ruiz Fernández M. D., Molero Jurado M. D. M. (2017). Inventory of overburden in alzheimer's patient family caregivers with no specialized training. *International Journal of Clinical and Health Psychology*.

[3] Santinello M. (2007). *Link Burnout Questionnaire Manuale*.

[4] Rodríguez-Mantilla J. M., Fernández-Díaz M. J. (2017). The effect of interpersonal relationships on burnout syndrome in secondary education teachers. *Psicothema*.

[5] Otero-López J. M., Castro C., Villardefrancos E., Santiago M. J. (2015). Job dissatisfaction and burnout in secondary school teachers: student’s disruptive behaviour and conflict management examined. *European Journal of Education and Psychology*.

[6] Shirom A., Quick J. C., Tetrick L. E. (2003). Job-related burnout: A review. *Handbook of Occupational Health Psychology*.

[7] Khani R., Mirzaee A. (2015). How do self-efficacy, contextual variables and stressors affect teacher burnout in an EFL context?. *Journal of Educational Psychology*.

[8] Klusmann U., Kunter M., Trautwein U., Lüdtke O., Baumert J. (2008). Engagement and emotional exhaustion in teachers: Does the school context make a difference?. *Applied Psychology*.

[9] Hultell D., Gustavsson J. P. (2011). Factors affecting burnout and work engagement in teachers when entering employment. *Work*.

[10] Gázquez J. J., Cangas A. J., Pérez-Fuentes M. C., Padilla D., Cano A. (2007). Percepción de la violencia escolar por parte de los familiares: un estudio comparativo en cuatro países europeos. *International Journal of Clinical and Health Psychology*.

[11] Llor-Esteban B., Sánchez-Muñoz M., Ruiz-Hernández J. A., Jiménez-Barbero J. A. (2017). User violence towards nursing professionals in mental health services and emergency units. *European Journal of Psychology Applied to Legal Context*.

[12] Skaalvik E. M., Skaalvik S. (2009). Does school context matter? Relations with teacher burnout and job satisfaction. *Teaching and Teacher Education*.

[13] García-Arroyo J. A., Osca A. (2017). Coping with burnout: Analysis of linear, non-linear and interaction relationships. *Anales de psicología*.

[14] Wheatley K. F. (2005). The case for reconceptualizing teacher efficacy research. *Teaching and Teacher Education*.

[15] Bandura A. (1977). Self-efficacy: toward a unifying theory of behavioral change. *Psychological Review*.

[16] Bandura A. (1997). *Self-Efficacy: The Exercise of Control*.

[17] Goddard R. D., Goddard Y. L. (2001). A multilevel analysis of the relationship between teacher and collective efficacy in urban schools. *Teaching and Teacher Education*.

[18] Goddard R. D., Hoy W. K., Hoy A. W. (2004). Collective efficacy beliefs:theoretical developments, empirical evidence, and future directions. *Educational Researcher*.

[19] Steca P., Picconi L., Gerbino M. (2002). Convinzioni di efficacia, percezioni di contesto e atteggiamenti verso il lavoro e soddisfazione. *Psicologia delleducazione e della formazione*.

[20] Elena B. P., Carmen T. U., Alicia A. M. (2010). Job satisfaction of secondary school teachers: effect of demographic and psycho-social factors. *Revista de Psicología del Trabajo y de las Organizaciones*.

[21] Evers W. J. G., Brouwers A., Tomic W. (2002). Burnout and self-efficacy: A study on teachers' beliefs when implementing an innovative educational system in the Netherlands. *British Journal of Educational Psychology*.

[22] Malinen O.-P., Savolainen H. (2016). The effect of perceived school climate and teacher efficacy in behavior management on job satisfaction and burnout: A longitudinal study. *Teaching and Teacher Education*.

[23] Jurado M. D. M. M., Pérez-Fuentes M. D. C., Linares J. J. G. G., Márquez M. D. M. S., Martínez Á. M. (2018). Burnout risk and protection factors in certified nursing aides. *International Journal of Environmental Research and Public Health*.

[24] Ventura M., Salanova M., Llorens S. (2015). Professional self-efficacy as a predictor of burnout and engagement: The role of challenge and hindrance demands. *The Journal of Psychology: Interdisciplinary and Applied*.

[25] Pérez-Fuentes M. d., Simón-Márquez M. d., Molero-Jurado M. d., Barragán-Martín A. B., Martos-Martínez Á., Gázquez-Linares J. J. (2018). Inteligencia emocional y empatía como predictores de la autoeficacia en Técnicos en Cuidados Auxiliares de Enfermería. *Revista Iberoamericana De Psicología Y Salud*.

[26] Jurado M. D. M. M., Pérez-Fuentes M. D. C., Linares J. J. G., Martín A. B. B. (2018). Burnout in health professionals according to their self-esteem, social support and empathy profile. *Frontiers in Psychology*.

[27] Durán M. A., Extremera N., Montalbán F. M., Rey L. (2005). Engagement y burnout en el ámbito docente: Análisis de sus relaciones con la satisfacción laboral y vital en una muestra de profesores. *Revista de Psicología del Trabajo y de las Organizaciones*.

[28] Esteras J., Chorot P., Sandín B. (2014). Predicción del burnout en los docentes: Papel de los factores organizacionales, personales y sociodemográficos. *Revista de Psicopatología y Psicología Clínica*.

[29] Platsidou M. (2010). Trait emotional intelligence of greek special education teachers in relation to burnout and job satisfaction. *School Psychology International*.

[30] Ruiz Quiles M., Moreno-Murcia J. A., Vera Lacárcel J. A. (2018). Del soporte de autonomía y la motivación autodeterminada a la satisfacción docente. *European Journal of Education and Psychology*.

[31] Mattern J., Bauer J. (2014). Does teachers' cognitive self-regulation increase their occupational well-being? The structure and role of self-regulation in the teaching context. *Teaching and Teacher Education*.

[32] Brackett M. A., Palomera R., Mojsa-Kaja J., Reyes M. R., Salovey P. (2010). Emotion-regulation ability, burnout, and job satisfaction among british secondary-school teachers. *Psychology in the Schools*.

[33] Judge T. A., Watanabe S. (1993). Another look at the job satisfaction-life satisfaction relationship. *Journal of Applied Psychology*.

[34] Tait M., Padgett M. Y., Baldwin T. T. (1989). Job and life satisfaction: a reevaluation of the strength of the relationship and gender effects as a function of the date of the study. *Journal of Applied Psychology*.

[35] Skaalvik E. M., Skaalvik S. (2017). Still motivated to teach? A study of school context variables, stress and job satisfaction among teachers in senior high school. *Social Psychology of Education*.

[36] Skaalvik E. M., Skaalvik S. (2010). Teacher self-efficacy and teacher burnout: A study of relations. *Teaching and Teacher Education*.

[37] Bermejo-Toro L., Prieto-Ursúa M., Hernández V. (2016). Towards a model of teacher well-being: personal and job resources involved in teacher burnout and engagement. *Journal of Educational Psychology*.

[38] Pérez-Fuentes M. M., Molero M. M., Gázquez J. J., Oropesa N. F. (2018). The role of emotional intelligence in engagement in nurses. *International Journal of Environmental Research and Public Health*.

[39] Martos Á., Pérez-Fuentes M. d., Molero M. d., Gázquez J. J., Simón M. d., Barragán A. B. (2018). Burnout y engagement en estudiantes de Ciencias de la Salud. *European Journal of Investigation in Health, Psychology and Education*.

[40] Preacher K. J., Hayes A. F. (2004). SPSS and SAS procedures for estimating indirect effects in simple mediation models. *Behavior Research Methods, Instruments, & Computers*.

[41] Preacher K. J., Hayes A. F. (2008). Asymptotic and resampling strategies for assessing and comparing indirect effects in multiple mediator models. *Behavior Research Methods Instruments & Computers*.

